# Adipose-Derived Stem Cells Respond to Increased Osmolarities

**DOI:** 10.1371/journal.pone.0163870

**Published:** 2016-10-05

**Authors:** Urška Potočar, Samo Hudoklin, Mateja Erdani Kreft, Janja Završnik, Krešimir Božikov, Mirjam Fröhlich

**Affiliations:** 1 Educell Ltd., Trzin, Slovenia; 2 Institute of Cell Biology, Faculty of Medicine, University of Ljubljana, Ljubljana, Slovenia; 3 Department of biochemistry and molecular biology, Jozef Stefan Institute, Ljubljana, Slovenia; 4 Department of Plastic Surgery and Burns, Division of Surgery, University Medical Centre Ljubljana, Ljubljana, Slovenia; National Centre for Scientific Research-Demokritos, GREECE

## Abstract

Cell therapies present a feasible option for the treatment of degenerated cartilaginous and intervertebral disc (IVD) tissues. Microenvironments of these tissues are specific and often differ from the microenvironment of cells that, could be potentially used for therapy, e.g. human adipose-derived stem cells (hASC). To ensure safe and efficient implantation of hASC, it is important to evaluate how microenvironmental conditions at the site of implantation affect the implanted cells. This study has demonstrated that cartilaginous tissue-specific osmolarities ranging from 400–600 mOsm/L affected hASC in a dose- and time-dependent fashion in comparison to 300 mOsm/L. Increased osmolarities resulted in transient (nuclear DNA and actin reorganisation) and non-transient, long-term morphological changes (vesicle formation, increase in cell area, and culture morphology), as well as reduced proliferation in monolayer cultures. Increased osmolarities diminished acid proteoglycan production and compactness of chondrogenically induced pellet cultures, indicating decreased chondrogenic potential. Viability of hASC was strongly dependent on the type of culture, with hASC in monolayer culture being more tolerant to increased osmolarity compared to hASC in suspension, alginate-agarose hydrogel, and pellet cultures, thus emphasizing the importance of choosing relevant *in vitro* conditions according to the specifics of clinical application.

## Introduction

Degeneration of cartilaginous tissues is a serious health problem, which affects a large percentage of the worldwide population. Only low back pain affects up to 85% of people during their lives and therefore represents a high social, healthcare, and economic burden [1, 2].

Cell therapies represent a possible approach for the treatment of intervertebral disc (IVD) and cartilage degeneration [3, 4, 5]. Human adipose-derived stem cells (hASC) have gained significant interest as a cell source due to their accessibility, limited donor site damage, high proliferation rate, and differentiation potential [5, 6, 7, 8, 9, 10, 11, 12]. Human adipose-derived stem cells can, in the form of high cell density three-dimensional (3D) cultures and in the presence of specific growth factors, such as BMP-7 and TGF-β, differentiate towards a chondrogenic phenotype and produce a proteoglycan-rich matrix [13, 14, 15, 16]. The use of hASC in cartilage [10, 14, 17, 18, 19] and IVD tissue engineering [17, 20, 21, 22, 23] has therefore been the subject of numerous *in vitro* and *in vivo* studies.

Specific microenvironmental conditions in the cartilage [24, 25] and the IVD are characterized by acidity, limited nutrition, low glucose, low oxygen concentrations and increased osmolarity [26, 27]. Osmotic swelling pressure is a consequence of the proteoglycan-rich matrix, which is one of the main characteristics of the functional nucleus pulposus and cartilage. The extracellular osmolarity in a healthy tissue ranges between 350–450 mOsm/L in the cartilage [26, 27] and 450–550 mOsm/L in the IVD [28, 29].

In the process of cell therapy implementation or *in vitro* studies, cells may be kept in various culture types, such as suspension (e.g. cell isolation from tissue or trypsinization), two-dimensional (2D) monolayer culture (e.g. cell expansion), or 3D scaffolds (e.g. for achieving conditions that support chondrogenic differentiation). In clinical practice, cells can be implanted in the form of suspension [22, 30, 31, 32] or embedded in 3D scaffolds [33, 34].

To ensure safe and efficient cell cartilage and IVD therapy, the implanted cells have to be able to survive at the implantation site, and moreover, need to produce an appropriate proteoglycan-rich matrix. As hASC are not exposed to increased osmolarities in their native tissue–osmolarity of lipoaspirate is approximately 315 mOsm/L [35], it is of great importance to understand if changes in osmolarity affect their phenotype and whether different culture types influence the cells’ response.

Increased osmolarity has been reported to cause dissimilar effects including a decrease [23, 27, 36] or increase [16, 37, 38, 39] in chondrogenic differentiation in various cell types (nucleus pulposus cells, chondrocytes, and mesenchymal stem cells) and culture conditions. Increased osmolarities of 485 and 500 mOsm/L have been shown to inhibit proliferation and viability [15, 23, 36] and have been reported to cause either a decrease or an increase of the chondrogenic potential of hASC in two different previous works [23] [15], in comparison to approximately 300 mOsm/L–- i.e. the osmolarity of the standard cell growth media for mammalian cells.

The aim of our study was therefore to investigate the effect of a broader range of cartilaginous tissue-specific osmolarities (400 mOsm/L–600 mOsm/L) on the viability, proliferation rate, morphology, and chondrogenic potential of hASC. Moreover, different culture types were compared with respect to their ability to support the viability of hASC upon exposure to increased osmolarities.

## Materials and Methods

### A. Isolation and culture of hASC

#### Isolation and expansion of hASC

The approval and written informed consent of the National Medical Ethics Committee of the Republic of Slovenia were obtained for the use of protocols and patient lipoaspirates, which were obtained from abdominal fat that represents waste material resulting from medical liposuction procedures (approval number 21/09/07). Research was conducted according to the principles expressed in the Declaration of Helsinki. Human adipose-derived stem cells were isolated using the procedure described by Zuk et.al. [6] and expanded in culture media. The cells were seeded at a density of 4000 cells/cm^2^ and expanded at 37°C with 5% CO_2_. Cells of the third and fourth passage were used for differentiation and viability/proliferation experiments, respectively. Three biological samples (donors) were used for all experiments, unless otherwise stated. For each biological sample several technical repeats (parallels) were performed. To avoid donor-specific responses, comparison of hASC viability in different culture types was performed on the same biological sample.

#### Monolayer culture

After 3 days, media were changed to media with different osmolarities. Subsequently, cells were assessed for viability, actin filament organization, nuclear changes, cell area, cell shape index (CSI), cell culture morphology, and proliferation. After the initiation of experiments, the cells were maintained in culture without subculturing for up to 4 weeks.

#### Suspension

Cells were expanded in monolayer cultures, trypsinized, pelleted, and re-suspended in the respective media with different osmolarities at a cell density of 1.6 x 10^6^ cells/mL. Viability of cells in suspension was then assayed.

#### Alginate-agarose hydrogel culture

Cells were expanded in culture media, trypsinized, rinsed with buffer without Ca2+, and counted. The cells were embedded in alginate-agarose hydrogel cylindrical constructs (dimensions: 10mm diameter × 2mm height) and solidified as previously described [40]. The hydrogels were transferred to culture media for 2 days and afterwards the respective media with different osmolarities were added. Hydrogel constructs were cut in half and the middle parts of the hydrogels were assessed for cell viability.

#### Pellet culture

To evaluate chondrogenic potential of hASC, cells were cultured in pellets. For preparation of each pellet, aliquots of 250,000 cells were centrifuged (500 x g, 10 min) and incubated at 37°C overnight in 1 mL of culture media (DMEM/F12, 10% fetal bovine serum (FBS, Gibco), 1% gentamycin). After 24 h, pellets were formed and culture media were changed to 300 mOsm/L media (of the same composition as culture media) or osmotic chondro-differentiation media. Pellets were processed for histology and histochemical analysis, upon which the viability and chondrogenic potential (acid proteoglycan content and compactness of pellets) were determined.

### B. Culture media

#### Culture media

Cells were routinely cultured in Dulbecco's modified eagle's media (DMEM/F12, Gibco) supplemented with 10% FBS, 1% gentamycin (0.05 mg/mL; Gibco), and basic fibroblast growth factor (bFGF, 1 ng/ml; Peprotech).

#### Media with different osmolarities

A sterile solution of 5 M NaCl (Sigma-Aldrich) and 0.4 M KCl (Sigma-Aldrich) was used to regulate the osmolarity of the culture media to the desired value. The osmolarities were adjusted to 308 ± 3 mOsm/L (control group), 401 ± 3 mOsm/L, 502 ± 2 mOsm/L, 600 ± 3 mOsm/L, and 903 ± 10 mOsm/L (Semi-Micro Osmometer K-7400, Knauer). Values for osmolarities are rounded to hundreds (300, 400, 500, 600, and 900 mOsm/L) for all media used in the study. Media adjusted to 900 mOsm/L served as an extreme, non-physiological control.

#### Osmotic chondro-differentiation media

Chondro-differentiation media were prepared according to the manufacturer’s protocol (Poietics™ human mesenchymal stem cells, Lonza) with minor modifications: DMEM/F12 (osmolarity: 301 mOsm/L) was used instead of differentiation basal media (osmolarity: 340 mOsm/L) to ensure all appropriate osmolarities (300, 400, and 500 mOsm/L). Additionally, two growth factors were added: TGF-β1 (10 ng/mL; PeproTech) and BMP-7 (100 ng/mL; PeproTech).

### C. Fluorescence experiments

A fluorescence microscope (Nikon, Eclipse T300) was used for visualization of fluorescent dye-stained samples.

#### Viability

For viability assessment, the Live/Dead staining kit (Invitrogen) was used following the manufacturer's protocol with modifications: incubation media were prepared in PBS with adjusted osmolarities, so that proper osmolarity values for each experimental group were maintained during the assay performance. Viability of cells was assayed at various time points: monolayer culture– 20 min (minutes), 1 h (hour), 24 h (hours), 4 d (days), and 4 w (weeks); suspension– 1 h and 24 h; hydrogel– 1 h, 24 h, and 4 d. Live and dead cells were counted and the viability was calculated by the following formula:

Viability (%) = (live cell count / total cell count) × 100.

#### Cell area and cell shape index

Digital images of the cells in monolayer culture stained with the Live/Dead kit were analyzed after 20 min, 1 h, 24 h, 4 d, and 4 w of exposure using Zeiss AxioVision software. The area of the cells was determined and the comparison was done between different osmolarities in time. To define cell shape index (CSI) the following formula was used: 4×π×A×P^-2^, A being the cell surface and P cell perimeter. CSI provides a measure of cell circularity: oriented (elongated) cells having values close to zero and circular cells having values closer to 1. CSI was calculated for 3 biological samples (40 technical repeats for each).

#### Nuclear changes and actin filament organization

Phalloidin (Sigma Aldrich) was applied to visualize actin cytoskeletal organization of the cells after 20 min, 1 h, 24 h, and 4 d. Fixation and staining were performed according to the manufacturer’s protocol. For the detection of nuclear changes, the same samples were stained with the DAPI nucleic acid stain (Vector laboratories).

### D. Scanning electron microscopy

Scanning electron microscopy (SEM) was used to analyze the samples that were prepared as previously described [41] and examined with a scanning electron microscope (Jeol JSM 840A) at time points 24 h, 14 d, and 4 w. Vesicle positive cells have been determined per surface area of 0,35 mm^2^ in all experimental groups and their absolute numbers were counted.

### E. Estimation of the proliferation rate of hASC

The proliferation rate of hASC in monolayer culture was evaluated after 4 d of growth in increased osmolarities. Cells were trypsinized and a hemocytometer and Trypan blue (Sigma Aldrich) were used to count live and dead cells. The initial number of seeded cells and the cell number after 4 d of culture were used to calculate population doublings (PD) using the formula:

PD = [log_10_(final number of cells)–log_10_(number of seeded cells)]/log_10_ [42].

### F. Assessment of the chondrogenic potential of hASC

To evaluate the chondrogenic potential of hASC, histological and histochemical analyses of pellets were performed after 4 w of culture. Pellets were washed in PBS and fixed in 4% paraformaldehyde in PBS overnight. Samples were embedded in paraffin, sectioned to 7–8 μm, and mounted on glass slides. The sections were deparaffinized with xylol and hydrated with a graded series of ethanol washes. To identify apoptotic cells, TUNEL staining was performed using the ApoptTag Peroxidase *In Situ* apoptosis detection Kit (Millipore) according to the manufacturer`s protocol. To assess the acid proteoglycan content, staining with Alcian blue (AB; Sigma Aldrich) was performed. The Fast Red (FR; Sigma Aldrich) and Hematoxylin (Merck Millipore) & Eosin Y (Merck Millipore) stain (HE) were used to assess pellet compactness. AB stains acid proteoglycans light blue, FR stains nucleic acids red and the cytoplasm pale pink, and HE stains nuclei violet blue and the cytoplasm pink.

### G. Statistical analysis

The GraphPad Prism (version 6.0) software was used for statistical analyses. Data are presented as means ± SD (standard deviation). Statistical significance was estimated by using one-way ANOVA and post-hoc tests for multiple comparisons among groups. A level of significance of p < 0.05 was used.

## Results

### Increased osmolarities affect viability, morphology, and proliferation of hASC in monolayer culture

#### Viability

**Fig 1:**
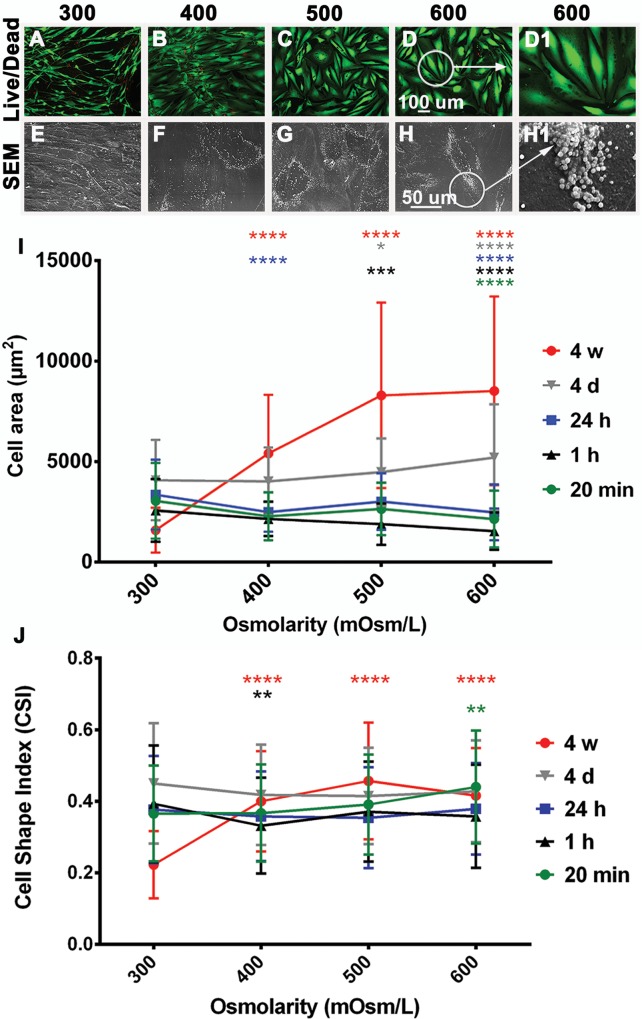
Viability and morphology of hASC after exposure to different osmolarities in monolayer culture. Cells were cultured in monolayer for up to 4 w in 300 mOsm/L (A, E), 400 mOsm/L (B, F), 500 mOsm/L (C, G) and 600 mOsm/L (D, H) media. Cells remained viable after 4 w under all osmolarities tested (Live/Dead assay: live cells are green, dead cells are red; A–D, D1 –higher magnification of the insert indicated in D). Three biological samples were used (2 technical repeats), one representative sample is shown.Morphology was assessed with SEM after 4 w (E–H). Numerous vesicles on the plasma membrane of cells (arrow) were detected in hASC cultures under increased osmolarities (F–H, H1 – higher magnification of the insert indicated in H). One biological sample was used (4 technical repeats), representative images are shown. Quantification of cell area and CSI based on Live/Dead images was performed for time points: 20 min, 1 h, 24 h, 4 d, and 4 w (I, J). Cell areas and CSI of different experimental groups (400, 500, and 600 mOsm/L) were compared to control group (300 mOsm/L) for each time point. Cell area and CSI values exhibited the most obvious increase after 4 w in response to increased osmolarities. Three biological samples were used for analysis. Means ± SD of 40 repeats are shown. * p = 0.0286; ** p = 0.0016; *** p = 0.0002; **** p < 0.0001

Human adipose-derived stem cells cultured in 400 mOsm/L, 500 mOsm/L, and 600 mOsm/L remained attached and had a comparable viability to that of cells cultured in 300 mOsm/L up to 4 w (Fig 1A–1D). Exposure of hASC to 900 mOsm/L allowed survival of cells for 1 h.

#### Morphology. Cell area and CSI

Quantification of cell areas based on the Live/Dead assay stain showed that cell areas changed in response to increased osmolarities, with the most prominent change at the longest time point of 4 w, where increased cell sizes were observed with all increased osmolarities (400, 500, and 600 mOsm/L) in comparison to the control (300 mOsm/L) with the following values: 300 mOsm/L (1590 μm^2^ ± 1115), 400 mOsm/L (5413 μm^2^ ± 2913), 500 mOsm/L (8297 μm^2^ ± 4615), and 600 mOsm/L (8521 μm^2^ ± 4690) (Fig 1I).

Increased osmolarities caused a spherical appearance which is consistent with the affect on cell area. The CSI exhibited the most prominent change after 4 w of culture: CSI showed higher values with 400 mOsm/L (0.4 ± 0.14), 500 mOsm/L (0.46 ± 0.16), and 600 mOsm/L (0.42 ± 0.13) in comparison to 300 mOsm/L (0.22 ± 0.09) (Fig 1J).

#### Morphology of cell culture

Vesiculation was triggered in response to increased osmolarities as observed by SEM (Fig 1F–1H). While in 300 mOsm/L media, vesicles were not observed, increased osmolarity of 400, 500, and 600 mOsm/L caused vesicle formation. Vesicle-positive cells were counted per surface area of 0,35 mm^2^ and numbers were as follows: (i) time point 24 h: 300 (0), 400 (0), 500 (0), 600 (35 vesicle positive cells); (ii) time point 14 d: 300 (0), 400 (24), 500 (24), 600 (31); (iii) time point 4 w: 300 (0), 400 (31), 500 (41), 600 (37). Statisticaly significant differences have been observed for all increased osmolarities (400, 500 and 600 mOsm/L) in comparison to 300 mOsm/L (*** p = 0.0001) for the time points 14 d and 4 w. At time point 24 h only 600 mOsm/L was statisticaly significant different to control 300 mOsm/L (*** p = 0.0002). One biological sample was used for analysis (4 technical repeats were used). In part, the increase in vesicle-positive cells in prolonged culture (14 d and 4 w) might also be due to cell proliferation.

#### Nuclear changes and actin filament organization

**Fig 2:**
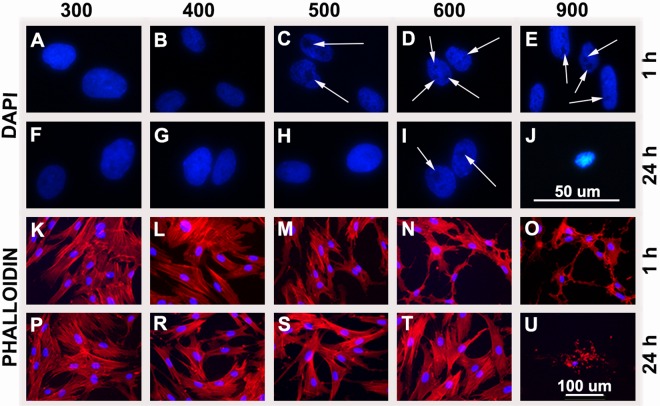
Nuclear changes and actin filament organization in hASC after exposure to increased osmolarities. Cells were cultured in monolayer in 300 mOsm/L (A, F, K, P), 400 mOsm/L (B, G, L, R), 500 mOsm/L (C, H, M, S), 600 mOsm/L (D, I, N, T) and 900 mOsm/L (E, J, O, U). Nuclear changes were assessed with DAPI after 1 h (A–E) and 24 h (F–J) of exposure. Unstained regions appeared after 1 h at values 500 mOsm/L (C), 600 mOsm/L (D) and 900 mOsm/L (E). After 24 h unstained regions were observed only with 600 mOsm/L (I). With 900 mOsm/L most of the cells detached and died. (Nucleus = blue; no DNA staining = black spots indicated by arrows). Actin filament organization was assessed after 1 h (K–O) and 24 h (P–U) of exposure to increased osmolarities. Differences in actin filament organization were observed after 1 h in groups 500 mOsm/L (M), 600 mOsm/L (N) and 900 mOsm/L (O) in comparison to 300 mOsm/L (K). No changes in actin filament organization were detected after 24 h of exposure under all tested osmolarities, except with 900 mOsm/L where most of the cells died and detached. (Actin fibers = red; nuclei = blue). For all experiments three biological samples were used.

Homogenous DAPI staining was characteristic for nuclei in hASC exposed to 300 (Fig 2A and 2F) and 400 mOsm/L (Fig 2B and 2G); however, it was comparable in all groups after 20 min of exposure (S1A–S1E Fig). The first intranuclear regions with no nucleic acid staining were present in hASC after 1 h of exposure to 500, 600, and 900 mOsm/L (Fig 2C, 2D and 2E). While this effect was reversed after 24 h of exposure to 500 mOsm/L (Fig 2H), it remained present in hASC in 600 mOsm/L (Fig 2I). However, after 4 d no nuclear changes were observed with osmolarities 300–600 mOsm/L (S1P–S1T Fig).

Actin organization was comparable in all groups (except for 900 mOsm/L) after 20 min (S1A1–S1E1 Fig) and it remained unchanged for hASC cultured in 400 mOsm/L (Fig 2L and 2R) in comparison to 300 mOsm/L after 1 h and 24 h (Fig 2K and 2P). First changes occurred after 1 h with 500, 600, and 900 mOsm/L (Fig 2M, 2N and 2O), where actin filaments were unusually formed. However, there were no more signs of actin filaments disruption after 24 h (Fig 2S and 2T) as well as after 4 d (S1P1–S1T1 Fig) of exposure with 500 and 600 mOsm/L.

#### Proliferation

**Fig 3:**
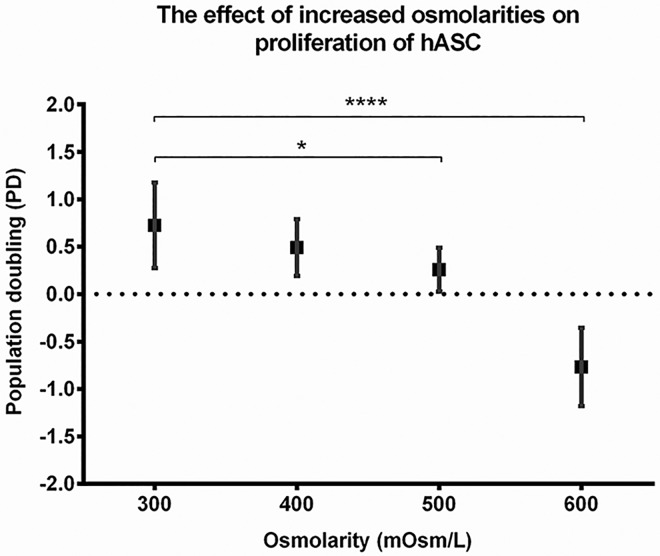
Proliferation of hASC in monolayer culture after exposure to different osmolarities. Population doublings are presented for hASC cultured under 300, 400, 500, and 600 mOsm/L after 4 d of culture (method: hemocytometer count, Trypan blue). A higher PD signifies a higher population rate. The dotted line represents the initial situation (day 0). Three biological samples were used. Means ± SD of 12 repeats are shown. * p = 0.04; **** p < 0.0001

The proliferation rate of hASC was reduced by increased osmolarities of 400 (population doubling; PD = 0.5), 500 (PD = 0.3), and 600 mOsm/L (PD = – 0.8) compared to 300 mOsm/L (PD = 0.7) after 4 d in monolayer culture (Fig 3).

### The viability of hASC in suspension and alginate-agarose hydrogel decreases with time

**Fig 4:**
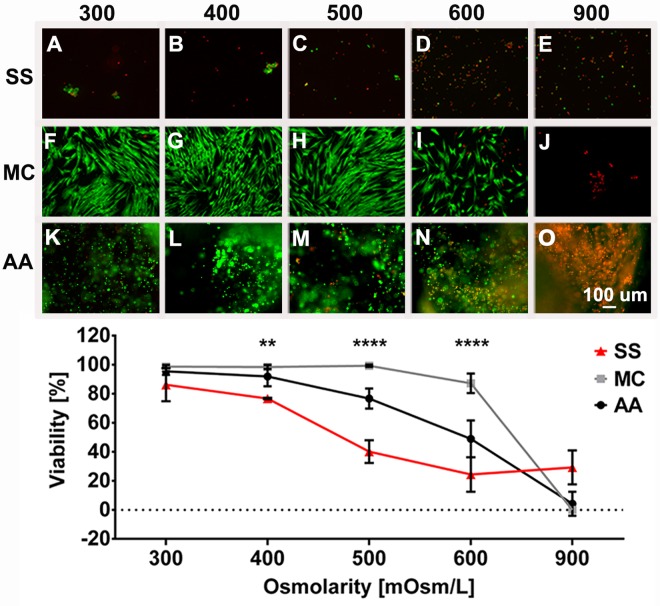
**Viability of hASC in suspension (SS), monolayer culture (MC) and alginate-agarose hydrogel (AA).** Cells were cultured for 24 h in 300 mOsm/L (A, F, K), 400 mOsm/L (B, G, L), 500 mOsm/L (C, H, M), 600 mOsm/L (D, I, N) and 900 mOsm/L media (E, J, O) in suspension (SS; A–E), monolayer (MC; F–J), and alginate-agarose hydrogel (AA; K–O). Live/Dead assay was performed (A–O; live cells are green, dead cells are red). Quantification of images A–O is presented in the graph. The comparison between different culture types was done on the same biological sample. For statistical analysis we compared the viability of all culture types within the same osmotic group. Means ± SD are presented. ** p = 0.0028; **** p < 0.0001

The viability of hASC in suspension culture decreased with time: there was a slight decrease in all groups after 1 h (up to 10%). However, after 24 h, increased osmolarities caused decreased viabilities in comparison to 300 mOsm/L; viabilities were as follows: 87% (300 mOsm/L), 77% (400 mOsm/L), 41% (500 mOsm/L), 24% (600 mOsm/L), and 29% (900 mOsm/L) (Fig 4A–4E, and corresponding graph; S2 Fig).

The viability of hASC decreased in alginate-agarose hydrogel culture with time. No negative impact on cell viability was observed after 1 h of exposure to all different osmotic conditions tested. While 24 h with 300 and 400 mOsm/L enabled a high cell viability of around 90%, 500, 600, and 900 mOsm/L resulted in significantly decreased viabilities and were: 77%, 49% and 4%, respectively (Fig 4K–4O, and corresponding graph; S2 Fig). After 4 d the viability still remained unchanged in 300 mOsm/L (95%), but it gradually decreased with increasing osmolarities: 84% (400 mOsm/L), 67% (500 mOsm/L), 38% (600 mOsm/L), and 5% (900 mOsm/L) (S2 Fig).

### Increased osmolarities reduce the chondrogenic potential of hASC in pellet cultures

**Fig 5:**
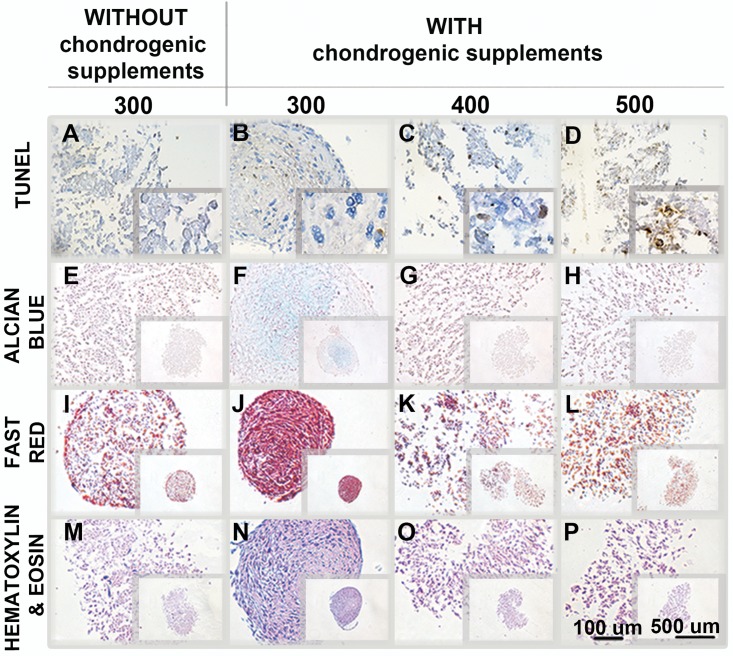
Chondrogenic differentiation of hASC in pellets. Pellets were cultured for 4 w in 300 mOsm/L media without chondrogenic supplements (A, E, I, M), 300 mOsm/L chondro-differentiation media (B, F, J, N), 400 mOsm/L chondro-differentiation media (C, G, K, O), and 500 mOsm/L chondro-differentiation media (D, H, L, P). Dead cells were determined by the TUNEL stain (A–D). Only rare dead cells (brown) were present in pellets cultured in 300 mOsm/L media (A, B). The numbers of dead cells increased with 400 mOsm/L (C), and were very frequent with 500 mOsm/L (D). The presence of acid proteoglycans (E–H) was assessed by Alcian Blue (acid proteoglycans = light blue). The structure and compactness of pellets (I–P) was assessed by Fast Red (nuclei = red, cytoplasm = pale pink) (I–L) and Hematoxylin & Eosin (nuclei = violet blue, cytoplasm = pink) (M–P). Three biological samples were used, one representative sample is shown.

In order to evaluate chondrogenesis of hASC in hyperosmotic conditions, the pellet cultures were established and the viability, acid proteoglycans content, and pellet structure were determined after 4 w of culturing in hyperosmotic media (Fig 5). The viability of cells was affected with increasing osmolarities: while 300 mOsm/L osmolarity resulted in only sparse dead cells, the number of dead cells increased with 400 mOsm/L, and were very frequent with 500 mOsm/L (Fig 5A–5D). Pellets cultured in 300 mOsm/L media without chondrogenic supplements (Fig 5A, 5E, 5I and 5M) exhibited a loose pellet structure and no acid proteoglycan deposition was detected. On the contrary, the addition of chondrogenic supplements to the 300 mOsm/L media resulted in compact acid proteoglycan-rich pellets (Fig 5B, 5F, 5J and 5N). Pellets cultured in 400 (Fig 5C, 5G, 5K and 5O) and 500 mOsm/L (Fig 5D, 5H, 5L and 5P) media supplemented with chondrogenic supplements were devoid of acid proteoglycans, exhibited poor structure with less extracellular matrix, and were looser, even compared to pellets in 300 mOsm/L media without chondrogenic supplements.

## Discussion

Human adipose-derived stem cells are the subject of many studies focusing on their chondrogenic and anti-inflammatory [43, 44, 45] potential in the context of clinical applications for cartilaginous tissue repair [13, 17, 18, 43, 46, 47, 48, 49]. However, the strikingly different physiology of the two tissues, namely metabolically highly active adipose tissue with endocrine function, where hASC derive from, and low metabolically active cartilaginous tissue as a host tissue, opens the question whether a new environment might affect the survival and viability of the implanted cells. Not only does the microenvironment itself differ among tissues and consequently represent a potentially sub-optimal condition for the host cells, but also cells within different tissues might be differentially regulated by the same trigger, such as pH, osmolarity, glucose, and oxygen [50].

One of the main characteristics of a functional nucleus pulposus and cartilage tissue is the proteoglycan-rich matrix, which results in increased extracellular osmolarity. Proteoglycan content varies with cartilaginous tissue type and zone [26, 27], mechanical load on the tissue [51], and age and degeneration stage [52], and therefore osmolarity values vary between 350–550 mOsm/L [26, 27, 28, 29].

In this study, we investigated how increased osmolarities in the range of relevant cartilaginous tissue values (400–600 mOsm/L) affect hASC in respect to their viability, morphological properties, proliferation rate, and chondrogenic potential.

### Impact on morphological changes and decreased proliferation of hASC as a result of increased osmolarities in monolayer culture

High resistance to hyperosmolarity is known for cells residing in organs associated with osmoregulation (e.g. kidney [53]) and was also described for endothelial cells [54, 55]. However, some cell types are sensitive to hyperosmolarity [36], which may also have an impact on cell proliferation [23, 36].

Several morphological changes of hASC were observed as a response to the hyperosmotic environment. In contrast to 300 mOsm/L where cells appeared fibroblast-like and stretched out, increased osmolarities caused an increase in size and spherical appearance (Fig 1A–1D). A similar spherical appearance was observed in human articular chondrocytes in response to increased osmolarities [39].

The cell area of hASC changed in response to increased osmolarities (Fig 1I). Xu and colleagues [37] demonstrated that the cell area was reduced in bovine articular chondrocytes cultured for up to 12 d in alginate beads. The volume of cultured chondrocytes was the largest at 280 mOsm/L and the lowest at 550 mOsm/L [37]. The extracellular osmotic environment affects cellular functions and also regulates cell volume [37], but might have different effects on different cell types and culture conditions. Based on the quantification of Live/Dead images, a reduction in cell size was noticed after 20 min and 1 h in response to increased osmolarities (Fig 1I). However, after 4 w an obvious increase in cell area was noticed with higher osmolarities (400, 500, and 600 mOsm/L) in comparison to 300 mOsm/L (Fig 1I). Shrinkage of cells after 1 h was also indicated by the actin stain (Fig 2M, 2N and 2O) with restoration of normal cell area after 24 h (Fig 2S and 2T). This is in accordance with a previous report, where human fibroblasts exhibited a reduction in cell area in response to hypertonic stress, but restored their normal size again after 3 h [56].

The formation of vesicles on the surface of hASC (Fig 1F–1H) is in accordance with the observation that changes in osmolarity result in reversible vesicle formation in synthetic giant unilamellar vesicles [57]. Since vesicles formed only in cells growing at higher osmolarities, vesicle formation due to paraformaldehyde fixation can be excluded [58]. This study demonstates that vesicles are formed already after 24 h and are still present after 14 d and 4 w, indicating a constitutive and non-reversible nature of vesicle formation. As the cell viability remains unchanged, vesicle formation might imply an adaptation of hASC to increased osmolarities in monolayer cultures.

Actin filament reorganization and changes in nuclear structure were detected in hASC with 500 and 600 mOsm/L after 1 h, and were returned to normal after 24 h (Fig 2M, 2S; 2N and 2T), which indicates that hASC are able to recover and can adapt to increased osmolarities. DNA damage and chromatin alterations due to hyperosmolarity have been reported for chondrocytes [59] and nucleus pulposus cells [60]. However, the osmosensing mechanism of cells enables the detection and repair of damage as wel as adaptation to hyperosmotic conditions during prolonged culture [59, 60]. Hyperosmolarity may also cause changes in the distribution of the nucleolar protein nucleolin [61], which inhibits DNA replication and prevents nucleolin from facilitating ribosomal biogenesis.

Proliferation of hASC in monolayer culture was negatively affected by increased osmolarities in a dose-dependant manner (Fig 3). Inhibition of proliferation was also demonstrated in previously published studies on hASC [15, 23], rat MSC (mesenchymal stem cells) [36], human articular chondrocytes [39, 62], and nucleus pulposus cells [63] in which increased osmolarity most probably influenced cell proliferation via the p38 MAPK and ATM-p53-p21^WAF1^-pRb pathways [60]. The observed decrease in hASC proliferation might also be related to nucleolin relocalization [61].

### hASC viability in response to osmolarities is culture type-specific

As cell viability is a prerequisite for safe therapy, we investigated the effects of increased osmolarities on hASC viability, which was not negatively affected by osmolarities from 300 to 600 mOsm/L (Fig 1A–1D), but was drastically decreased with 900 mOsm/L.

During the preparation phase and the cell therapy itself, cells might experience several different environments: cells might be implanted in the form of cell suspension [22, 30, 31, 32] and subsequently attach to the surrounding tissue, or might be implanted along with a scaffold, that is 2D or 3D [64]. As cells can respond differently to the same trigger when maintained in different culture types [65, 66], it is important to understand how a different culture types influence the cells’ response. We showed that hASC viability was differently affected by increased osmolarities in different cell culture types: hASC cultured in 2D monolayer culture were the most resistant to hyperosmolarity with unchanged viabilities in all groups (400–600 mOsm/L) up to 4 w. In contrast, hASC in suspension were the most affected–the viability was significantly reduced by increased osmolarities after 24 h, where less than 41% and 24% of hASC survived with 500 and 600 mOsm/L, respectively. Alginate-agarose hydrogel serves as a cell carrier for clinical applications and is a commonly used scaffold for providing a 3D environment in *in vitro* studies [33, 34, 40]. It is therefore necessary that cells survive on hydrogel carriers under hyperosmotic conditions. However, this study has demonstrated a significant decrease in cell viability (of 3D hydrogel cultures) after 24 h and 4 d with 500 and 600 mOsm/L. On the contrary, collagen II hydrogen-embedded hASC showed no reduction in viability at 500 mOsm/L up to 21 d in the culture [15].

Comparing all three culture types tested (monolayer culture, suspension, and hydrogel), 1 h of exposure to increased osmolarities did not negatively affect cells in any of the culture types (S2 Fig). While viability was not affected after 24 h in monolayer cultures, it was reduced in hydrogel culture and lowest in suspension (Fig 4 and S2 Fig), demonstrating that the osmolarity-induced decrease in viability strongly depends on the culture type. Prolonged culture of 4 d still did not notably affect viability in monolayer culture, but did further reduce the viability of cells on hydrogel (S2 Fig). The observed differential response of hASC to different environments thus underlines the importance of selecting appropriate experimental set-ups according to the specifics of the clinical application.

### Reduction of chondrogenic differentiation potential of hASC under increased osmolarities

For the successful progression of engineered cartilage, it is necessary to determine which culture conditions support successful extracellular matrix synthesis. Pellet cultures and chondrogenic supplements supported chondrogenesis of hASC with 300 mOsm/L. Osmolarity of 400 mOsm/L caused–despite the presence of chondrogenic factors in the culture media–a reduction in hASC viability, acid proteoglycan and extracellular matrix production, and inferior pellet compactness compared to 300 mOsm/L chondrogenic or non-chondrogenic media (Fig 5). Pellets cultured in 500 mOsm/L had a significant portion of dead cells present, thus the absence of acid proteoglycan production was most probably a consequence of the absence of live cells. The decrease in chondrogenic differentiation potential is in accordance with findings that matrix production is sensitive to osmotic stress [27, 67]. A decrease in gene expression of cartilage-related matrix proteins was also shown on chondrogenically non-induced rat MSC [36] and hASC [23] at 485 mOsm/L in monolayer culture. When hASC were embedded and cultured in 3D hydrogels, hyperosmolarity decreased the amount of glycosaminoglycans (GAGs) within the 3D hydrogels and increased GAGs in the media, indicating hindered retention of GAGs within the hydrogel cultures and thus increased release of GAGs into the surrounding culture media. On the contrary, increased osmolarity (380 mOsm/L) significantly increased the expression of the chondrogenic markers Col2a1, Col10a1, Acan, Sox9, Runx2 and GAGs of human bone marrow stem cells on gene and protein levels [38]. Also human chondrocytes [39], bovine and human IVD cells [16, 68], and bovine articular chondrocytes [37, 69] exhibited enhanced expression of chondrogenic markers in hyperosmotic conditions.

Differences in the response to hyperosmolarity among studies can be attributed to the different experimental set-ups used, including: cell types, culture types, culture conditions, sources for osmolarity adjustment (NaCl/KCl, sucrose, urea, sorbitol, etc.) and different methods and analyses [70].

In summary, hASC respond in a dose- and time-dependent fashion to increased osmolarities with decreased viability, proliferation, chondrogenic differentiation potential, and morphological changes, including changes in cell area, vesicle formation, actin cytoskeletal changes and morphological changes of nuclei. Cells are differentially susceptible to increased osmolarities in different culture types: while monolayer culture enables cell survival under increased osmolarities for up to 4 w in culture, a negative impact on hASC viability in suspension and in hydrogel culture is noticed already after 24 h. The observed culture type-specific differential response of hASC to increased osmolarity emphasizes the importance of selecting the appropriate experimental set-up: namely, taking into account tissue-specific conditions (e.g. specific osmolarities) as well as culture types (e.g. suspension, monolayer culture, 3D culture) relevant for clinical application when studying the potential of hASC for cartilaginous tissue repair in vitro.

## Supporting Information

S1 Fig Nuclear changes and actin filament organization in hASC after exposure to increased osmolarities. Cells were exposed to increased osmolarities in monolayer culture for 20 min, 1 h, 24 h and 4 d. Nuclear changes were assessed with DAPI (A-U). There were no changes after 20 min of exposure (A-E). Intranuclear regions with no nucleic acid staining (indicated by arrows) were first noticed in hASC after 1 h with 500 mOsm/L (H), 600 mOsm/L (I) and 900 mOsm/L (J). The effect was reversed after 24 h of exposure to 500 mOsm/L (M), but remained present in hASC with 600 mOsm/L (N). However, after 4 d no nuclear changes were observed under osmolarities 300–600 mOsm/L (P, R, S, T). (Nucleus = blue; no DNA staining = black spots indicated by arrows). Actin filament organization was assessed after exposure to increased osmolarities (A1–U1). There were no changes after 20 min and the first differences in actin filament organization were observed after 1 h with 500 mOsm/L (H1), 600 mOsm/L (I1), and 900 mOsm/L (J1) in comparison to 300 mOsm/L (F1). No changes in actin filament organization were detected after 24 h (K1, L1, M1, N1) or 4 d (P1, R1, S1, T1) of exposure under all tested osmolarities except for 900 mOsm/L where most of the cells died and detached (O1, U1). (Actin fibers = red; nuclei = blue). For all experiments three biological samples were used.
(TIF)

S2 Fig Viability of hASC in suspension (SS), monolayer culture (MC) and alginate-agarose hydrogel (AA) at different time points after exposure to different osmolarities. Cells of all culture types were exposed to increased osmolarities at the same time points 1 h, 24 h, and 4 d (time point 4 d for SS was not performed). Live/Dead assay was performed and quantification of viability is presented in the graph. The comparison of hASC viability in different culture types was performed on the same biological sample to avoid donor-specific responses. For statistical analysis, we compared the viability of all culture types (SS, MC, AA) within one time point. Means ± SD of 4 repeats are presented. There were no statisticaly significant differences in viability between SS, MC, and AA after 1 h of exposure. On the contrary, there were statisticaly significant differences after prolonged exposures (of 24 h and 4 d). Blue asterisks—differences between SS, MC, and AA after 24 h of exposure; black asterisks—differences between MC and AA after 4 d of exposure. **** p < 0.0001
(TIF)
