# Supplementary figures and images for: Adipose-Derived Stem Cells Respond to Increased Osmolarities
Source: PLoS One. 2016 Oct 5;11(10):e0163870. doi: 10.1371/journal.pone.0163870 (PMC5051864; doi:10.1371/journal.pone.0163870)

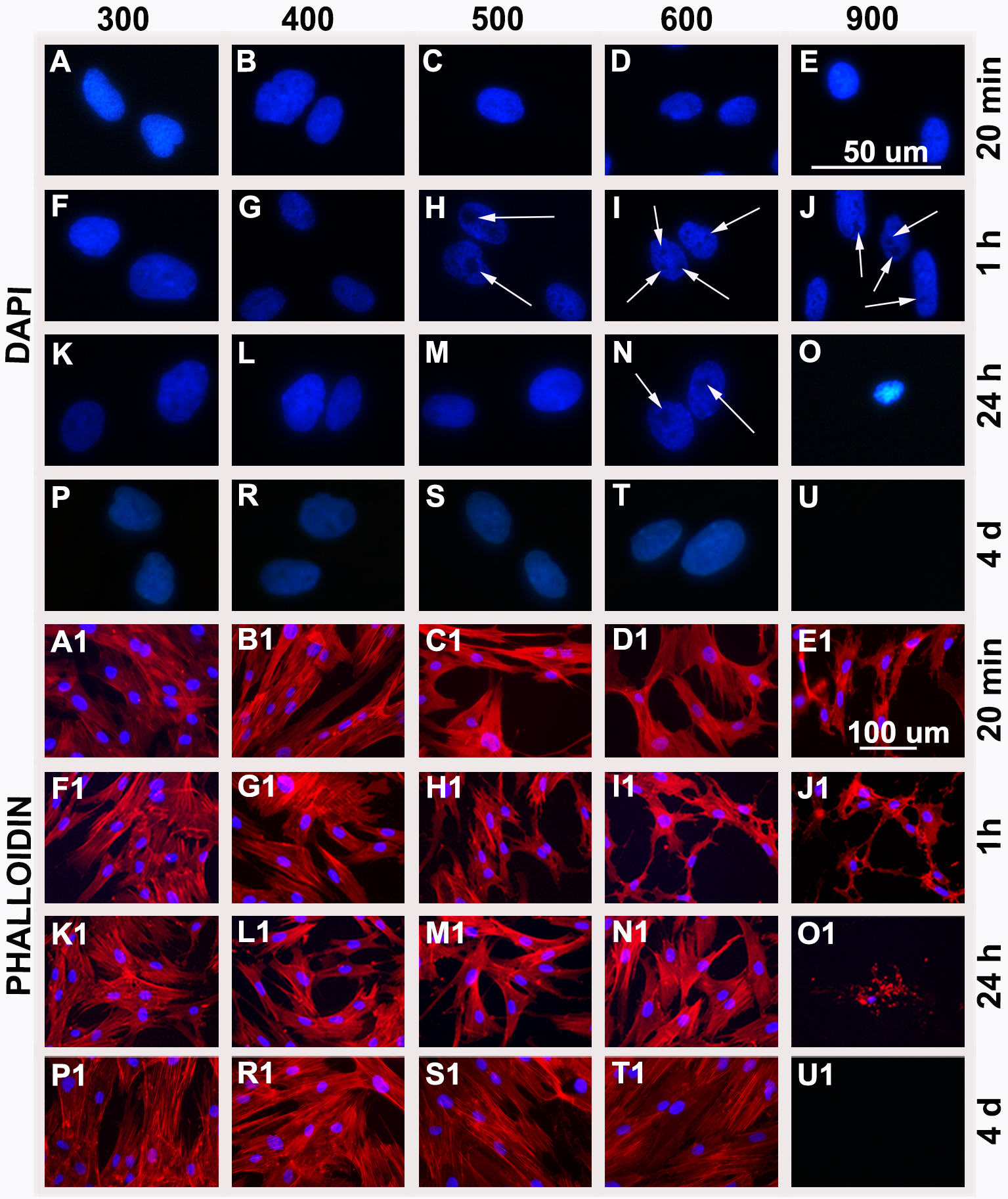

Supplement: S1 Fig — Cells were exposed to increased osmolarities in monolayer culture for 20 min, 1 h, 24 h and 4 d. Nuclear changes were assessed with DAPI (A-U). There were no changes after 20 min of exposure (A-E). Intranuclear regions with no nucleic acid staining (indicated by arrows) were first noticed in hASC after 1 h with 500 mOsm/L (H), 600 mOsm/L (I) and 900 mOsm/L (J). The effect was reversed after 24 h of exposure to 500 mOsm/L (M), but remained present in hASC with 600 mOsm/L (N). However, after 4 d no nuclear changes were observed under osmolarities 300–600 mOsm/L (P, R, S, T). (Nucleus = blue; no DNA staining = black spots indicated by arrows). Actin filament organization was assessed after exposure to increased osmolarities (A1–U1). There were no changes after 20 min and the first differences in actin filament organization were observed after 1 h with 500 mOsm/L (H1), 600 mOsm/L (I1), and 900 mOsm/L (J1) in comparison to 300 mOsm/L (F1). No changes in actin filament organization were detected after 24 h (K1, L1, M1, N1) or 4 d (P1, R1, S1, T1) of exposure under all tested osmolarities except for 900 mOsm/L where most of the cells died and detached (O1, U1). (Actin fibers = red; nuclei = blue). For all experiments three biological samples were used. (TIF) [file pone.0163870.s001.tif]

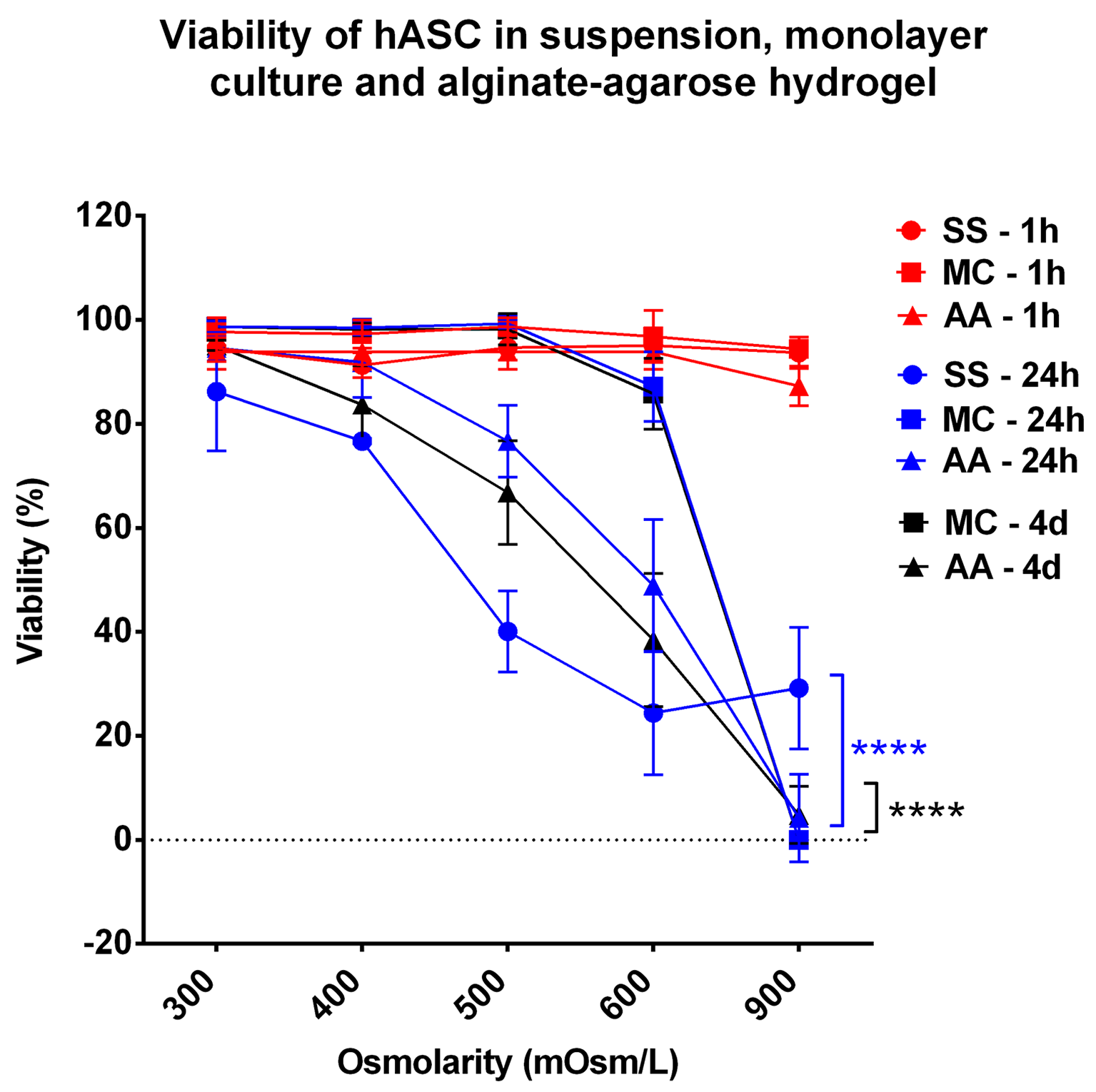

Supplement: S2 Fig — Cells of all culture types were exposed to increased osmolarities at the same time points 1 h, 24 h, and 4 d (time point 4 d for SS was not performed). Live/Dead assay was performed and quantification of viability is presented in the graph. The comparison of hASC viability in different culture types was performed on the same biological sample to avoid donor-specific responses. For statistical analysis, we compared the viability of all culture types (SS, MC, AA) within one time point. Means ± SD of 4 repeats are presented. There were no statisticaly significant differences in viability between SS, MC, and AA after 1 h of exposure. On the contrary, there were statisticaly significant differences after prolonged exposures (of 24 h and 4 d). Blue asterisks—differences between SS, MC, and AA after 24 h of exposure; black asterisks—differences between MC and AA after 4 d of exposure. **** p < 0.0001 (TIF) [file pone.0163870.s002.tif]
